# Three maxims for countering sex essentialism in scientific research

**DOI:** 10.1186/s13293-025-00748-x

**Published:** 2025-10-28

**Authors:** Marion Boulicault, Annika Gompers, Lauren Aalami, Ann Caroline Danielsen, Emily C. Dore, Patricia Homan, Katharine M. N. Lee, Miriam Miyagi, Hannah Niederriter, Atlas Sanogo, Maayan Sudai, Alex Thinius, Sarah S. Richardson

**Affiliations:** 1 Philosophy Department, University of Edinburgh, 50 George Square, Edinburgh, EH8 9LH UK; 2 Emory University Rollins School of Public Health, 201 Dowman Dr, Atlanta, GA 30322 USA; 3 Stanford University School of Medicine, 291 Campus Drive Li Ka Shing Building, Stanford, CA 94305 USA; 4 Department of Social and Behavioral Sciences, Harvard T.H. Chan School of Public Health, 677 Huntington Avenue, Boston, MA 02115 USA; 5 Department of Sociology, Florida State University, 222 S Copeland St, Tallahassee, FL 32306 USA; 6 Department of Anthropology, Tulane University, 6823 St Charles Ave, New Orleans, LA 70118 USA; 7 Center for Computational Molecular Biology, Brown University, 70 Brown St., Providence, RI 02912 USA; 8 Committee on Degrees in the Study of Women, Gender, and Sexuality, Harvard University, Boylston Hall, Cambridge, MA 02138 USA; 9 Faculty of Law, University of Haifa, Abba Khoushy Ave 199, 3498838 Haifa, Israel; 10 Department of Linguistics and Literature, Universiteit Van Amsterdam, 1012 WP Amsterdam, Netherlands; 11 Department of the History of Science, Harvard University, Science Center, Cambridge, MA 02138 USA

## Abstract

To explain observed disparities in health outcomes between men and women, sex essentialist approaches assign causal primacy to sex-related biology. In this essay, we present three case studies to illustrate how sex essentialism can distort human biomedical research and distill three maxims for countering this distortion: (1) engage in responsible citation practices; (2) generate and weigh alternative hypotheses for apparent observations of sex differences; (3) take care in constructing the appropriate denominator when making sex comparisons. We offer these maxims as broadly applicable standards of evidence to guide biomedical research that includes analysis of potential sex differences, as well as to support Institutional Review Boards (IRBs), funders, publishers, and peer reviewers in evaluating sex difference findings. If widely applied, these maxims would substantially improve the rigor, precision, and utility of the knowledge base of sex and gender science.

**Supplementary Information:**

The online version contains supplementary material available at 10.1186/s13293-025-00748-x.

## Highlights

- Sex essentialism distorts human biomedical research by assigning causal primacy to sex-related factors and ignoring how gender-related factors impact biology
- To illustrate strategies for countering sex essentialism, we offer case studies of sex differences in COVID-19, adverse drug events, and anterior cruciate ligament injury
- Engaging in responsible citation practice can combat the uncritical repetition of sex difference claims that embed sex essentialism in human biomedical research
- Generating and weighing alternative hypotheses for apparent sex differences found in data can reveal the important role of gender-related social factors in shaping human biology
- Constructing appropriate denominators for making sex comparisons can help account for the gendered social processes that shape data generating mechanisms

**Supplementary Information:**

The online version contains supplementary material available at 10.1186/s13293-025-00748-x.

## Introduction

Findings of biomedical sex difference research are routinely underpowered, unreplicated, or uninterpretable, as several recent reviews demonstrate [1–4]. While these problems have long plagued sex difference science [5–8], they have become newly salient in recent years in the context of institutional mandates such as the Sex as a Biological Variable (SABV) policy of the US National Institutes of Health (NIH) and the recently-released UK-based Medical Science Sex and Gender Equity (MESSAGE) framework. Both of these mandates require researchers to “consider sex” by including female models and presenting data separately for female- and male-classified groups [9, 10]. These policies have unleashed a firehose of sex difference claims, often interpreted as evidence of fundamental sex differences rooted in biology, even as such reports are grounded in study designs inadequate to support these claims [1]. Although sex inclusion policies were intended to improve biomedical research and address androcentric bias [11], systemic problems in the knowledge ecology of sex differences research threaten rigor, precision, and replicability in the field [2, 12–16].

The persistent challenges facing sex difference science cannot be resolved solely through improved statistical methods or more empirical evidence. The fundamental issue is conceptual: enduring *sex essentialism* [2, 17] in research design and interpretation. To characterize sex essentialism, we begin with some definitions. Sex is conventionally defined as “the different biological and physiological characteristics of males and females, such as reproductive organs, chromosomes, hormones, etc.,” and gender as “the socially constructed characteristics of women and men—such as norms, roles and relationships of and between groups of women and men” [18]. Recognizing that sex and gender interact, the term “gender/sex” has emerged to describe how “gender and sex cannot be easily or at all disentangled” [19].

Biological essentialism refers to theories and frameworks that account for differences between categories by “ascribing to them enduring, fixed, and prototypical biological characteristics” [20]. Biological essentialism is often connected to or justified by the closely related theory of biological determinism, which holds that “human behavior originates in and is dictated by biological entities or processes” [20]. Evolutionary biologist and social theorist Richard Lewontin describes biological essentialism and determinism together as a system of explanation that attempts to “deal with observed variation in human social conditions by an appeal to the determinative role of individual biology” [21]. Sex essentialism is a subset of biological essentialism that assigns causal primacy to sex-related biology in explaining disparities between women and men. As such, sex essentialist approaches to designing research studies, gathering and analyzing data, and interpreting and translating results largely focus on average differences between binary sex-groups. Any findings of difference are then used to make generalizations about sex-related biological causal pathways, including predictions about how men and women, and males and females, differ in their baseline range of capacities, vulnerabilities, preferences, and tendencies.

Sex essentialism ignores how gender-related social factors affect biological outcomes. In so doing, sex essentialism distorts human biomedical research. For example, a variable may *correlate* with sex in a population without being actually *caused* by it. As we discuss below, there are cases where mortality is higher among men as a group, but biological sex characteristics are not *solely* responsible for this disparity; and there are cases where sex essentialist models can obscure significant variation within, and overlap between, sex-groups. For biomedical researchers with a primary training in biological systems, it may be challenging to recognize gender as a meaningful source of variation, but the examples below demonstrate that considering the social dimensions of biological variation can be very significant in human biomedical research.

In addition to distorting scientific inference, sex essentialism produces harms to individuals and populations. Historically and in the present day, basic sex difference science is routinely cited to support, fuel, and sustain harmful systems of gender inequality, stigma, and stereotypes [22], as well as the exclusion of LGBTQ + people from human rights protections [23]. Unwarranted claims about biological sex differences have resulted in sex-based dosing practices that result in dangerous under- or overdosing, as in the case of Ambien [24], or an unfounded focus on developing and advertising specialized equipment to prevent Anterior Cruciate Ligament (ACL) injury in women [25] that allows gendered harms of underinvestment in women’s facilities, coaching, and training programs to continue [26]. As in the case of race essentialist bias in medicine and biology [27, 28], there is therefore an ethical and social responsibility for scientific researchers to not only avoid but to actively counter sex essentialism [29, 30].

In this essay, we present three case studies to illustrate how sex essentialism distorts science and produces harms: Adverse Drug Events, COVID-19, and Anterior Cruciate Ligament (ACL) Injury (Table 1). In each of these cases, which represent multiple fields of human biomedical science, essentialist inferences about biological sex differences are commonplace but not well founded. From these case studies, we distill three maxims for researchers exploring sex differences: (1) engage in responsible citation practices; (2), generate and weigh alternative hypotheses for apparent sex differences; (3) take care in constructing the appropriate denominator when making sex comparisons. We offer these maxims as broadly applicable standards of evidence to guide biomedical research that includes analysis of potential sex differences, as well as to support Institutional Review Boards (IRBs) [31], funders, publishers, and peer reviewers in evaluating sex difference findings. If widely applied, these three maxims would improve the rigor, precision, and utility of scientific research on sex, gender, and their interaction.

**Table 1:** Table illustrating how each maxim applies to each case study

	Maxim 1 (Citation Practices)	Maxim 2 (Alternative Hypotheses)	Maxim 3 (Denominators)
ADEs	The widespread claim that women experience twice the rate of ADEs compared to men is cited through layers of review papers that ultimately rest on a decades-old, methodologically-limited studies	Gender differences in healthcare seeking behaviors, medication usage, clinician treatment, and experience of events as adverse drive observed sex disparities in ADEs in pharmacovigilance databases, rather than women’s assumed biological vulnerability	Appropriately accounting for gender differences in medication usage, i.e., the population at risk of an ADE, renders ADE rates very similar among women and men
COVID-19	A highly publicized claim of sex differences in immune function underlying observed disparities in COVID-19 outcomes proliferated, with the subsequent dissensus rarely cited	Variation in observed sex disparities in COVID-19 outcomes over time, geography, and racialized group indicate the role of social-contextual factors rather than biological factors alone	Women’s higher rates of COVID-19 testing led to increased detection of mild cases, thereby making women’s case fatality rate appear smaller than that of men
ACL injuries	Clinical websites and academic literature uncritically report a 2–8 × greater risk for females without citation	Systemic gender differences in resources and environment for men’s and women’s sports—manifested as e.g., team size and training time—shape ACL injury risk, rather than biological sex-related factors alone	Smaller team sizes and lower proportionate amount of training time among women’s sports make women’s calculated exposure time smaller than men’s, thereby inflating women’s observed injury rates

## Case studies

The three examples presented below demonstrate how sex essentialism can bias scientific research as well as how biomedical scientists can avoid and counter this bias.

### Adverse drug events

The claim that women experience adverse drug events (ADEs) at up to twice the rate of men is widely cited as evidence that more research is needed on biological sex differences [9, 32, 33]. But research analyzing ADE reports in the US Food and Drug Administration Adverse Event Reporting System showed that, after accounting for differing prescription drug usage rates in men and women, apparent sex differences in ADEs are more likely to be close to zero [34]. This demonstrates that to accurately measure a sex difference (or lack thereof) it is imperative to use an appropriate denominator—in this case, the number of people using medications as opposed to the number of people in the general population, by gender.

Sex essentialism also has implications in the clinic, as illustrated by the FDA’s 2013 decision to halve the recommended dosage of the sleep aid zolpidem (trade name Ambien) for women. This decision is considered a case in point of the relevance of sex-based biology to human health, citing sex differences in drug metabolism. However, slower zolpidem clearance rates among women are rendered non-statistically significant after adjusting for body weight [24]. This means that the FDA’s recommended sex-based dosing leaves many women undertreated and men overtreated for debilitating sleep disorders [35].

Both of the above examples illustrate how sex essentialist biases can distort our understanding of sex disparities in reports of ADEs. But sex essentialist assumptions can also lead us to attribute sex differences in ADEs to biology quite generally. As Lee et al. [36] show, gender-related social and structural variables likely play a substantial role in generating higher reports of ADEs in women compared to men. This includes gendered differences in seeking healthcare and being prescribed a drug, gendered experiences of whether a drug-related event is adverse, and gender-based disparities in clinician reports of ADEs to surveillance databases. This suggests that explaining apparent sex disparities in ADEs requires generating alternative explanations that consider the influence of gendered social, structural and cultural factors on health outcomes, rather than biological sex-based factors alone.

### COVID-19 outcomes

In the early days of the COVID-19 pandemic, media and researchers reported that men were dying at higher rates than women, leading to a widespread sex essentialist assumption that innate sex-related biological factors contributed to COVID-19 risk. However, Gompers et al. [37] showed that the denominator (i.e. number of cases) used in the calculations of case fatality rates by gender/sex was influenced by women’s increased likelihood of getting tested for COVID-19 even when not sick, because of gendered norms surrounding health care use (i.e., women go to the doctor more often than men), gendered occupational structures and pregnancy care (i.e., women are overrepresented among nurses, frontline health workers, and pregnant people, who were frequently tested for COVID) [37–41]. This means that cases among women were more likely to be detected even if mild, whereas cases among men were less likely to be detected unless severe, artificially inflating the case fatality ratio among men.

In addition, research showed that in the US, gender/sex differences in case and mortality rates varied substantially across time and place [42, 43], as well as across racialized groups [44]. This variation would not be expected were gender/sex differences primarily attributable to immutable sex-related biological characteristics.

Finally, Shattuck-Heidorn et al. [45] highlighted that apparent sex differences in immune function [46] often disappeared when variables such as body mass index and age were taken into account. Neglecting to control for these factors has led to incorrect conclusions regarding the etiology of sex differences and to misguided public health interventions [45, 46].

The case of COVID-19 illustrates how sex essentialist frameworks fail to consider gendered structural factors that shape denominators of health metrics, variation in gender/sex differences over time and place, and important interacting variables such as body size and age, which together suggest that observed differences are not attributable to biological sex alone.

### ACL knee injury in athletes

It is commonly claimed that women athletes have a higher rate of ACL knee injury than men, and this disparity has been widely attributed to musculoskeletal and hormonal differences between the sexes [47–50]. This presumed biological vulnerability has undergirded concerns about the risks of women and girls’ participation in sport and has been used in the marketing of specialized gear and products for women designed to compensate for it [25, 51].

But numerous gender-related social factors influence the occurrence and reporting of ACL injuries [52], including well-documented and large disparities in resources such as equipment, playing surface, and coaching staff; activity history during development; gender norms about body image, dieting, and reporting pain; and responsibilities outside of sport, including caregiving and paid employment. Digging into the epidemiological research positing a 2–10 times higher rate of ACL injury in female athletes [49, 53, 54], Danielsen et al. [26] found that gendered sporting environments may have inflated this estimate. Specifically, the incidence of ACL knee injury is commonly calculated using an aggregate denominator that multiplies the number of team members by the total number of games and practices that occurred during a given time period, regardless of the time individual players actually spent on the playing field and without distinguishing between practice and training time. Because, for example, a history of deep social inequality in the domain of sport means that men's teams are, on average, larger than women's, the average woman player will have more high-risk match play than the average man. Yet, the athlete exposure denominator for women will be smaller than for men, artificially inflating estimates of women’s injury rates.

The case of ACL injury demonstrates how the assumption that disparities are due to biology has led sports and exercise science to overlook gendered social factors such as unequal training opportunities and sport resources. In addition, it again shows how biases in denominators can distort our understanding of observed sex disparities.

## Maxims for sex and gender science

From these case studies, we distill three maxims for best practices in countering biological sex essentialism in human biomedical research.

### Practice responsible citation

Uncritical citation of decontextualized sex difference findings and citational tunnels to outdated, tenuous sex difference claims are a problem for the evidence base of biomedical science that appeared in all three case studies discussed above. A particularly striking example is found in the case of adverse drug event rates, frequently reported to be twice as high among women compared to men in the scientific literature and in media outlets such as the *New York Times* [55]. However, the source of this oft-repeated 2:1 statistic is unclear: as shown in Supplementary Fig. 1, the citational chain reveals reliance on reiterations of the assertion in multiple layers of review papers that ultimately rest on a handful of decades-old studies with obvious methodological limitations.

In the case of ACL knee injury, sources ranging from academic articles to patient-facing medical materials regularly assert that women athletes experience ACL injuries at 2–10 times the rate of men. A Yale Medicine website states that this disparity is true “according to research,” without providing any citations. The Northwestern Medicine website does provide a link to a 2016 editorial in the *Journal of Orthopaedics*—but this article is focused on *why* “the female ACL” is “more prone to injury,” and (like the patient-facing sites) states as a given that “women tear the ACL two to eight times more frequently than men,” with no supporting references [49, 56, 57]. Based on a recent “State of the Art” review of sex differences in ACL injury as an anchor paper [47], it is evident that the empirical evidence for this statistic is also filtered through several review papers that almost all rely on the same empirical study of collegiate basketball and soccer published in 1995 [58] (Supplementary Fig. 2). Meanwhile, a recent meta-analysis of 58 studies found a rate ratio of 1.7, much closer to parity [59].

Tracking citation metrics in the case of COVID-19 also demonstrates the risk of selective and inaccurate citation practices. The paper by Takahashi et al. [46] purporting to find sex differences in immune responses to COVID-19 has been cited 1,562 times as of February 2025, whereas a critique demonstrating that the study’s findings do not support a sex difference claim [45]—also published in *Nature*—has been cited a mere 38 times. Years later, the sweeping claim that biological sex differences in immune function underlie observed differences in COVID-19 outcomes continues to be cited as a foundation for new research (e.g., [60, 61]), without noting critical debate and dissensus in the published literature immediately adjacent to the study.

Miscitation represents a spectrum of practices of relying on outdated, selective, inaccurate, or improperly contextualized claims of sex differences. Appropriate citation is a fundamental ethical and epistemic requirement of all scientific research, and—among other requirements—involves fully reading the studies one cites and citing the original source for a claim rather than secondary sources [62, 63]. Yet these standards are often not met in studies of sex differences, as we have described here. The uncritical citation of existing sex difference claims without ascertaining the origin and quality of the underlying evidence allows sex essentialism to become embedded in scientific research and biomedical practice and reified as a foundational assumption in risk metrics, data collection protocol, and prognostic tools [72].

### Generate and weigh alternative hypotheses

Scientists examining sex differences in human biomedical research are advised to generate, weigh, and when possible, test, alternative hypotheses in order to avoid defaulting to sex-essentialist explanations, as in the case studies discussed in this essay.

When evidence that men were dying at higher rates of COVID-19 emerged, many rushed to assert that biological differences between men and women were the cause, leading some to suggest sex-specific therapeutics for COVID-19 [46, 64–67]. Yet a closer look showed substantial variation in the magnitude and direction of gender/sex disparities in case and mortality rates across time, place, and racialized group [42–44], suggesting an important role for social-contextual factors. Furthermore, Shattuck-Heidorn et al. [45] showed that what were assumed to be sex-related differences in immune function were actually better explained by variables such as body mass index and age [45].

In the case of ACL injuries, close analysis reveals that the perceived sex gap in ACL injury rates, often attributed to innate biological differences, may be significantly influenced by structural factors such as differences in team sizes and relative training to match time between men's and women's sports [26]. These findings underscore the need to consider alternative explanatory frameworks to sex-based biological explanations, such as frameworks related to disparities in training quality and resource allocation.

Similarly, while sex disparities in ADE reports in pharmacovigilance databases are typically attributed to women’s biological vulnerability to ADEs, a vast evidence base supports the alternative hypothesis that gender-related social factors are the primary drivers: empirical analysis demonstrates that gender differences in healthcare utilization, bias in the clinic, experience of an event as “adverse,” and pre-existing structural determinants of health explain much of the observed disparities [36].

Generating and testing a range of hypotheses is vital to ensure that sex essentialist assumptions are thoroughly examined. The case studies illustrate two more specific strategies to generate and weigh alternative hypotheses: first, examining variation in sex disparities across time, place, and group, and second, considering how gender-related, structurally rooted factors could contribute to differences in the body between gender/sex categories.

### Mind the denominator

All three case studies show overstatement of the magnitude of sex differences due to inaccurately or poorly defined denominators.

In the case of ACL injury studies, the denominator in injury rates is an aggregated measure of time at risk for injury. However, the comparability of injury rate denominators by gender is undermined by factors connected to longstanding underinvestment in women's sports, such as smaller team sizes and less training time relative to matches compared to men. These factors generate smaller denominators for women and mischaracterize the distribution of individual injury risk, with the consequence of inflating injury rates among women.

Sex disparities in rates of ADEs offer another example. Due to gendered norms and health behaviors, women are much more likely to take medications than men, meaning that the number of women at risk of ADEs is larger. Appropriately accounting for rates of medication use by gender in the denominator reveals very similar rates of ADEs among women and men, contrary to the oft-repeated statistic that women experience ADEs at a rate of up to twice that of men.

Finally, in the case of COVID-19, gendered norms and occupations led women to be more likely to be tested for COVID-19 than men, leading to increased detection of mild or asymptomatic cases among women. The resulting smaller denominator among men, containing fewer and more severe cases, likely distorted the case fatality rate and made COVID-19 appear far more deadly for men than women [37].

When interpreting findings relevant to sex disparities in human health, researchers are encouraged to take into account the extensive evidence that gendered social processes shape behavior and data collection such that the resulting data may not be comparable across groups.

## Discussion

Today, an increasing number of biomedical researchers understand that attending to sex-related variables is required for inclusive science [68] and recognize that the exclusion of transgender, nonbinary, and intersex people from studies may limit the inclusiveness, accuracy and clinical applicability of findings [69]. These developments represent an advancement in methodological sophistication in sex difference research. Yet many fail to consider the potential for this turn towards sex difference science to produce unintended harm by amplifying sex essentialist assumptions in biomedical research and failing to account for the interaction of sex and gender.

To move the future of evidence in sex and gender science forward, there is a need both to accelerate the consideration of social variables in research on biomedical sex disparities and to address unfounded, misleading, and tangibly harmful inferences in sex differences science. Fundamental to this is countering biological sex essentialism by acknowledging how gender interacts with sex in human populations [70], often inextricably so [19].

To illustrate the importance of addressing sex essentialism by adhering to these maxims and building gender/sex interaction into the foundations of sex and gender science, we offer an anticipatory example of emerging research at the intersection of Alzheimer’s risk prediction, artificial intelligence (AI), and precision medicine. Precision medicine researchers propose developing sex-stratified models for Alzheimer’s Disease and Related Dementias (ADRDs) risk, which utilize sex categories as a source of predictive information through separate computational models and numerical cutoffs for each sex [71]. Without great care, attempts to develop and apply sex-stratified machine learning approaches to predict ADRD risk and progression may embed sex essentialist assumptions into neurocognitive knowledge and research programs [72]. For example, if successful, sex-specific predictive models will likely lead to sex essentialist inferences of innate biological differences between men and women in the brain and cognition. Yet this inference obscures how demographic factors such as lifespan and gendered social variables such as education, intimate partner violence, and socioeconomic status are known to play a large role in sex disparities in ADRDs [73–75]. If we do not move the needle on sex essentialism in biomedicine, sex essentialist assumptions can be unwittingly built into the precision medicine tools of the future.

Integrating social and biological complexities in human biomedical research is an ongoing challenge, with distinct dimensions across different research fields. Nonetheless, transformative work in areas ranging from heart disease [76] to pain research [77] to sperm counts [78] illustrates its potential to drive innovation and clinically relevant insight. In this essay, we offer real-world, teachable case studies showing the harms of biological sex essentialism, which can cause research to focus on biological determinants of sex differences at the cost of rigor and precision. Countering this bias in research on sex differences in human biomedicine, which are often shaped by gender-related social factors in interaction with sex-related ones, requires vigilance and care. We hope that these case studies and maxims can serve as touchstones for developing robust standards of evidence for documenting and reporting biomedical sex differences in sex and gender science, from the characterization of human health phenomena in preclinical laboratory models to studies of human populations.

## Supplementary Information

**Additional file 1: Figure S1** This citational tree traces the claim that women experience ADEs at twice the rate of men, using Zucker and Prendergast [33], cited 547 times as of February 2025, as an anchor paper [33]. Review papers are shaded in orange and original articles are shaded in blue. Zucker and Prendergat [33] state the 2:1 statistic as fact in the Introduction, providing three references, none of which support the claim. One of these references shows gender parity in ADEs in children reported to a French pharmacovigilance database [79], while one reports results of a questionnaire-based study [80], and one is a review of mechanisms underlying women’s greater risk of ADEs [81]. Upon investigation, the Tharpe [81] review similarly takes women’s twofold risk of ADEs as a given, citing two further reviews as supporting references [82, 83]. Reidl and Casillas do not provide an estimate of the sex disparity in ADEs, and simply mention “female gender” as one of the “specific factors that increase the risk of general adverse drug reactions,” citing yet another review [84]. We note that we were unable to access any of the three citations referenced in Barranco and Lopez-Serrano [84], all of which were in Spanish. Whitley and Lindsey [83] specify that women face a 50-75% increased risk of ADEs, which they, too, attribute to a review article in a dermatology journal [85]. Rademaker finally does include several original empirical citations to support the claimed 50-75% disparity, several of which investigate only a single type of ADE [86–89] or a single class of drug [90]. Other articles focus exclusively on patients admitted to a hospital or clinic for an ADE, and cannot account for users of a medication who experienced no or mild ADEs [91, 92]. Fattinger et al. [93] and Domecq et al., [94] did find slightly higher rates of ADEs among already hospitalized women compared to men [93, 94], and a meta-analysis by Martin et al. [95] did find that women were 1.6 times (95% CI 1.5-1.7) as likely as men to have an ADE reported to the UK’s Prescription Event Monitoring (PEM) surveillance system - however this system is known to be limited by incomplete and biased reporting [95, 96]. In addition to these specific methodological limitations, it should be noted that overall, the empirical evidence base underpinning the still-circulating 2:1 statistic is two to three decades old.

**Additional file 2: Figure S2** This citational tree traces the claim that women experience an ACL injury rate two to eight times that of men, using a recent “State of the Art” article [47] as an anchor paper. Review papers are shaded in orange and original articles are shaded in blue. Gianakos et al. [47] cite five papers for this claim. The first is an empirical article of collegiate athletes that found a sex disparity of 2.4 and 4.1 among soccer and basketball players, respectively [58]. This 30 year old article is highly cited - as of February 2025, it has been cited 2,538 times. Moreover, three of the citations used by Gianakos et al. 47 are themselves review papers that refer back to Arendt and Dick [48, 50, 53, 58]. Sutton and Bullock [48] cite only paper, a review [97], which again refers back to Arendt and Dick [58], as well as two empirical articles. One of these [98] reports the number of ACL injuries among male and female basketball players at a single college but does not provide the number of players on each team and therefore cannot calculate injury rates. The other [99] also studies collegiate basketball players, and finds a six times higher prevalence and eight times higher frequency of ACL injury among women compared to men. The final reference in Gianakos et al. 47 is an empirical study that does not quantify sex differences - rather, this analysis of 24 women soccer players finds that anatomical features were not associated with knee injuries [100]. The evidence base for women’s supposed two to eight times higher rate of ACL injury - as presented in this “State of the Art” review - therefore appears to rest entirely on analyses of two collegiate sports in the 1990s [58, 99].

## Data Availability

No datasets were generated or analysed during the current study.
