# Supplementary figures and images for: Three maxims for countering sex essentialism in scientific research
Source: Biol Sex Differ. 2025 Oct 28;16:83. doi: 10.1186/s13293-025-00748-x (PMC12560604; doi:10.1186/s13293-025-00748-x)

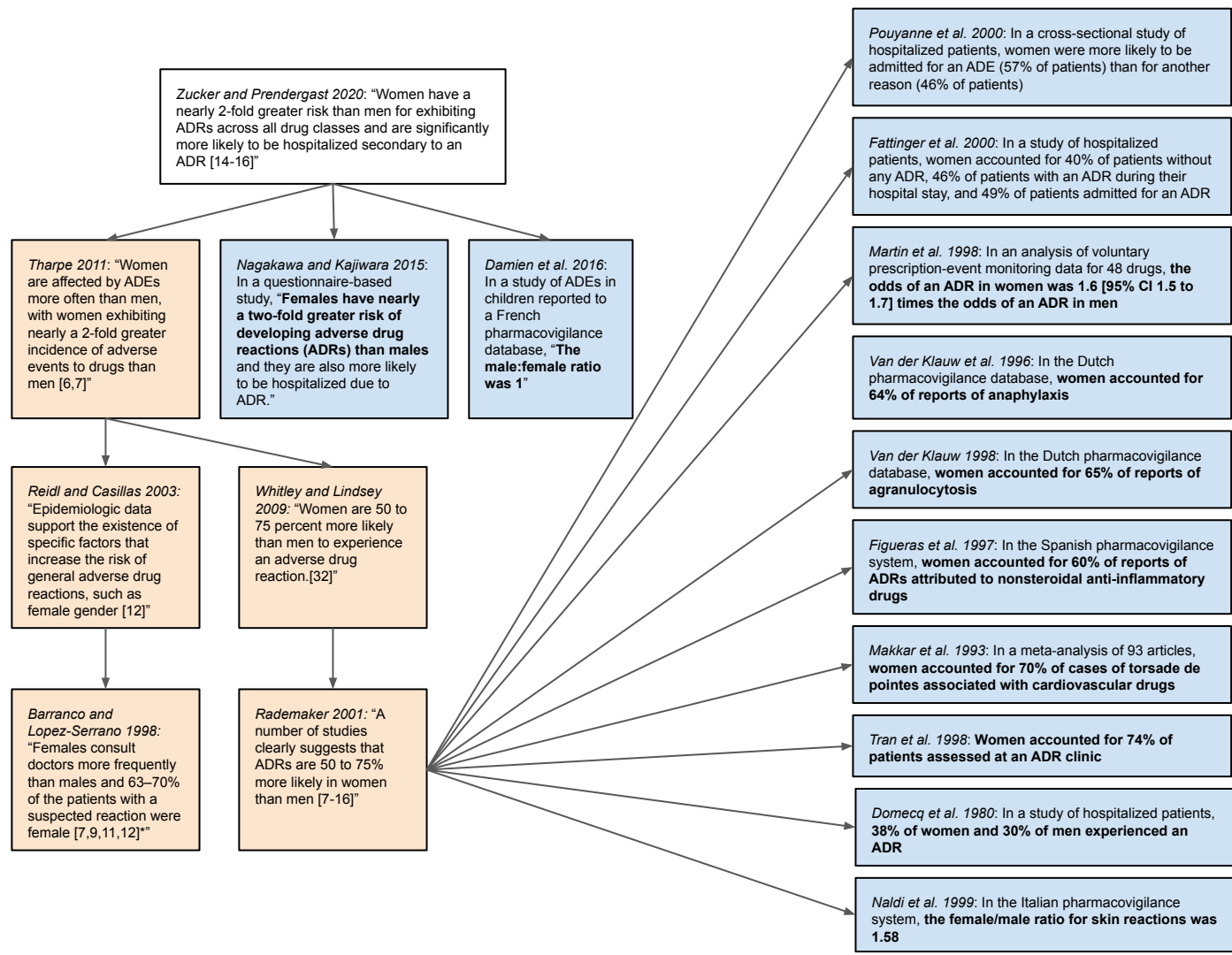

Supplement: Supplementary file 1 — Additional file 1: Figure S1 This citational tree traces the claim that women experience ADEs at twice the rate of men, using Zucker and Prendergast [33], cited 547 times as of February 2025, as an anchor paper [33]. Review papers are shaded in orange and original articles are shaded in blue. Zucker and Prendergat [33] state the 2:1 statistic as fact in the Introduction, providing three references, none of which support the claim. One of these references shows gender parity in ADEs in children reported to a French pharmacovigilance database [79], while one reports results of a questionnaire-based study [80], and one is a review of mechanisms underlying women’s greater risk of ADEs [81]. Upon investigation, the Tharpe [81] review similarly takes women’s twofold risk of ADEs as a given, citing two further reviews as supporting references [82, 83]. Reidl and Casillas do not provide an estimate of the sex disparity in ADEs, and simply mention “female gender” as one of the “specific factors that increase the risk of general adverse drug reactions,” citing yet another review [84]. We note that we were unable to access any of the three citations referenced in Barranco and Lopez-Serrano [84], all of which were in Spanish. Whitley and Lindsey [83] specify that women face a 50-75% increased risk of ADEs, which they, too, attribute to a review article in a dermatology journal [85]. Rademaker finally does include several original empirical citations to support the claimed 50-75% disparity, several of which investigate only a single type of ADE [86–89] or a single class of drug [90]. Other articles focus exclusively on patients admitted to a hospital or clinic for an ADE, and cannot account for users of a medication who experienced no or mild ADEs [91, 92]. Fattinger et al. [93] and Domecq et al., [94] did find slightly higher rates of ADEs among already hospitalized women compared to men [93, 94], and a meta-analysis by Martin et al. [95] did find that women were 1.6 times (95% [file 13293_2025_748_MOESM1_ESM.pdf]
