# Supplementary material for: Three maxims for countering sex essentialism in scientific research
Source: Biol Sex Differ. 2025 Oct 28;16:83. doi: 10.1186/s13293-025-00748-x (PMC12560604; doi:10.1186/s13293-025-00748-x)
Supplement: Supplementary file 2 — Additional file 2: Figure S2 This citational tree traces the claim that women experience an ACL injury rate two to eight times that of men, using a recent “State of the Art” article [47] as an anchor paper. Review papers are shaded in orange and original articles are shaded in blue. Gianakos et al. [47] cite five papers for this claim. The first is an empirical article of collegiate athletes that found a sex disparity of 2.4 and 4.1 among soccer and basketball players, respectively [58]. This 30 year old article is highly cited - as of February 2025, it has been cited 2,538 times. Moreover, three of the citations used by Gianakos et al. 47 are themselves review papers that refer back to Arendt and Dick [48, 50, 53, 58]. Sutton and Bullock [48] cite only paper, a review [97], which again refers back to Arendt and Dick [58], as well as two empirical articles. One of these [98] reports the number of ACL injuries among male and female basketball players at a single college but does not provide the number of players on each team and therefore cannot calculate injury rates. The other [99] also studies collegiate basketball players, and finds a six times higher prevalence and eight times higher frequency of ACL injury among women compared to men. The final reference in Gianakos et al. 47 is an empirical study that does not quantify sex differences - rather, this analysis of 24 women soccer players finds that anatomical features were not associated with knee injuries [100]. The evidence base for women’s supposed two to eight times higher rate of ACL injury - as presented in this “State of the Art” review - therefore appears to rest entirely on analyses of two collegiate sports in the 1990s [58, 99]. [file 13293_2025_748_MOESM2_ESM.pdf]

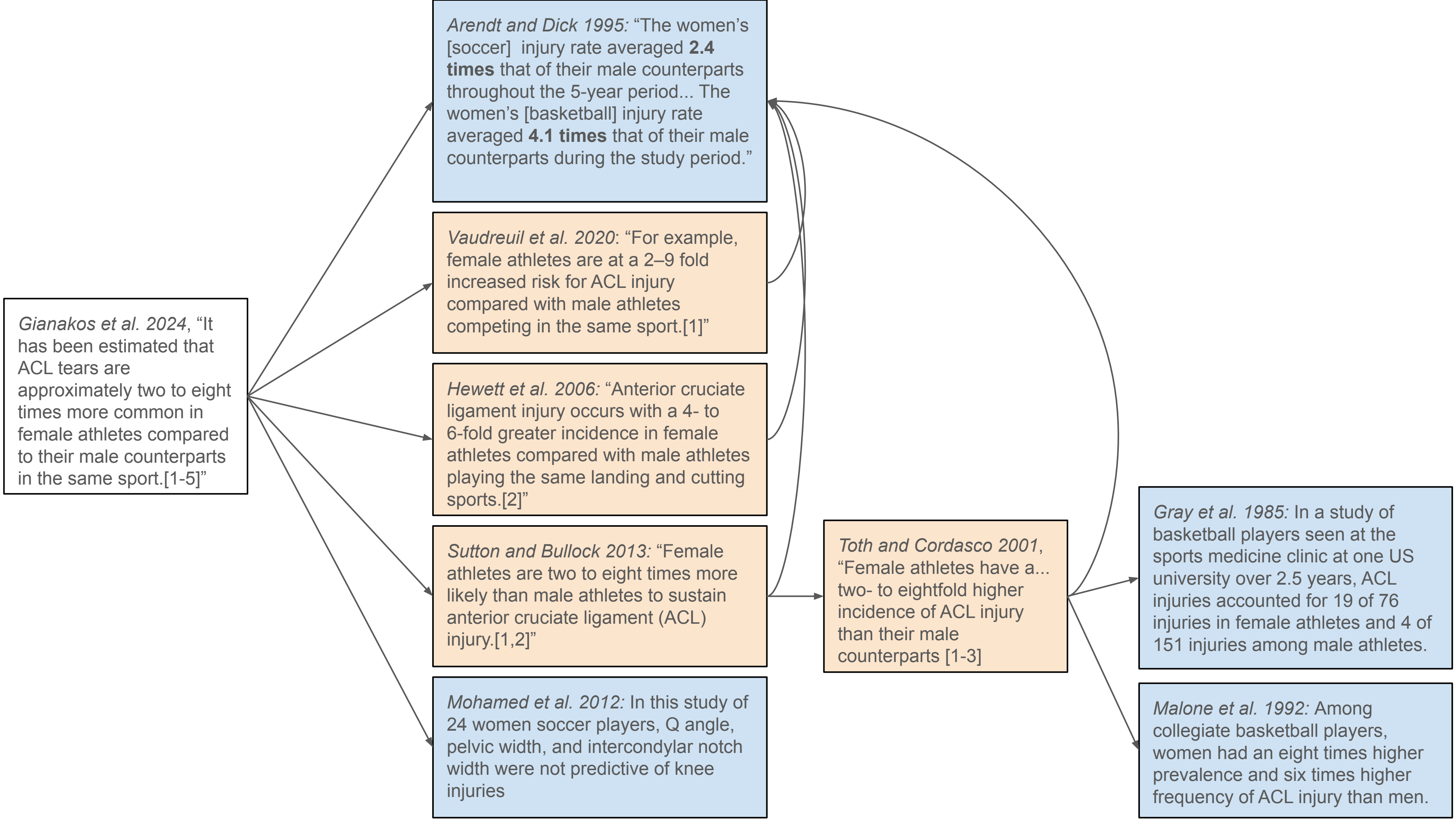
Gianakos et al. 2024, "It has been estimated that ACL tears are approximately two to eight times more common in female athletes compared to their male counterparts in the same sport.[1-5]"
Arendt and Dick 1995: "The women's [soccer] injury rate averaged 2.4 times that of their male counterparts throughout the 5-year period... The women's [basketball] injury rate averaged 4.1 times that of their male counterparts during the study period."
Vaudreuil et al. 2020: "For example, female athletes are at a 2–9 fold increased risk for ACL injury compared with male athletes competing in the same sport.[1]"
Hewett et al. 2006: "Anterior cruciate ligament injury occurs with a 4- to 6-fold greater incidence in female athletes compared with male athletes playing the same landing and cutting sports.[2]"
Sutton and Bullock 2013: "Female athletes are two to eight times more likely than male athletes to sustain anterior cruciate ligament (ACL) injury.[1,2]"
Mohamed et al. 2012: In this study of 24 women soccer players, Q angle, pelvic width, and intercondylar notch width were not predictive of knee injuries
Toth and Cordasco 2001, "Female athletes have a... two- to eightfold higher incidence of ACL injury than their male counterparts [1-3]
Gray et al. 1985: In a study of basketball players seen at the sports medicine clinic at one US university over 2.5 years, ACL injuries accounted for 19 of 76 injuries in female athletes and 4 of 151 injuries among male athletes.
Malone et al. 1992: Among collegiate basketball players, women had an eight times higher prevalence and six times higher frequency of ACL injury than men.
